# Synthesis and Evaluation of the Anti-Oxidant Capacity of Curcumin Glucuronides, the Major Curcumin Metabolites

**DOI:** 10.3390/antiox4040750

**Published:** 2015-12-02

**Authors:** Ambar K. Choudhury, Suganya Raja, Sanjata Mahapatra, Kalyanam Nagabhushanam, Muhammed Majeed

**Affiliations:** 1 Sami Labs Limited, Bangalore 560058, India; E-Mails: suganya@clinworld.org (S.R.); bioresearch@samilabs.com (S.M.); mail1@samilabs.com (M.M.); 2 Sabinsa Corporation-East Windsor, NJ 08520, USA

**Keywords:** curcumin monoglucuronide, curcumin diglucuronide, Synthesis, Anti-oxidant, DPPH, ORAC

## Abstract

Curcumin metabolites namely curcumin monoglucuronide and curcumin diglucuronide were synthesized using an alternative synthetic approach. The anti-oxidant potential of these curcumin glucuronides was compared with that of curcumin using DPPH scavenging method and Oxygen Radical Absorbance Capacity (ORAC) assay. The results show that curcumin monoglucuronide exhibits 10 fold less anti-oxidant activity (DPPH method) and the anti-oxidant capacity of curcumin diglucuronide is highly attenuated compared to the anti-oxidant activity of curcumin.

## 1. Introduction

Curcumin (**1**) is the major curcuminoid present among the naturally occurring three curcuminoids, long believed to be bioactive components, isolated from natural *Curcuma longa* Linn. (turmeric). Turmeric is mainly consumed as spice in India and Asia and also used in traditional medicine since ancient times.The unique structure of curcumin with its 1,3-diketo sub-structure, existing mainly in the enolic form in solution and exclusively in the solid state has created a pharmacological conundrum in that it interacts with diverse pharmacological targets without palpable toxicity even at fairly high doses. Pharmacological studies over the last few decades on curcumin have revealed its therapeutic potential as anti-cancer [1], anti-oxidant [2], anti-inflammatory [3], and antimicrobial [4] agents, and curcumin also possesses anti-arthritic [5], anti-diabetic [6], hepatoprotective [7], neuroprotectant [8] and antidepressant [9] activities.

The poor aqueous solubility and rapid metabolism of curcumin have often been cited as mitigating factors for the transition of curcumin into a more useful therapeutic agent [10]. The pleiotropic pharmacological activities of curcumin despite its metabolism have given rise to the speculation whether the *in vivo* pharmacological activities of curcumin are attributable through its metabolites. Curcumin metabolizes rapidly to several components. The major components of the Phase II metabolites are the glucuronides of curcumin and are assumed to occupy an important place in bioactivity of curcumin after its administration orally [11,12,13]. Some of the formulations of curcumin were reported to have enhanced bioavailability of curcumin actually did not enhance curcumin in plasma but only increased the concentration of metabolite, curcumin glucuronide [14,15]. Oral ingestion of these formulations in fact failed to detect any free curcumin in circulation. After administered orally, curcumin is invariably found in almost negligible quantity in plasma [14,15]. Despite its low concentration in plasma, curcumin still exhibits its biological activity even though no systematic study exists on the detection of curcumin in target tissues. Numerous studies [1,2,3,4,5,6,7,8,9,10,11,12,13,14,15,16] have been carried out on curcumin in last few decades to understand its mechanism of action; however, the low bioavailability and retention of bioactivity of curcumin still remain unexplained conundrums. Hence, to understand the mechanism of action of curcumin more precisely, it is essential to study the biological activities/functions of its metabolites too. Although one of the major metabolites of curcumin is found to be curcumin monoglucuronide [16,17], when curcumin is administered orally, the biological activity of curcumin monoglucuronide could not be studied previously due to the lack of availability of these molecules. There are many reports on the identification of curcumin glucuronides in plasma by LC-MS [17,18,19], however only in 2014, two groups [20,21] separately have synthesized curcumin glucuronides enzymatically [20] and chemically [21] in reasonable quantities and studied its biological activity on certain human cancer cell lines and reported the bioactivity.

**Figure 1:**
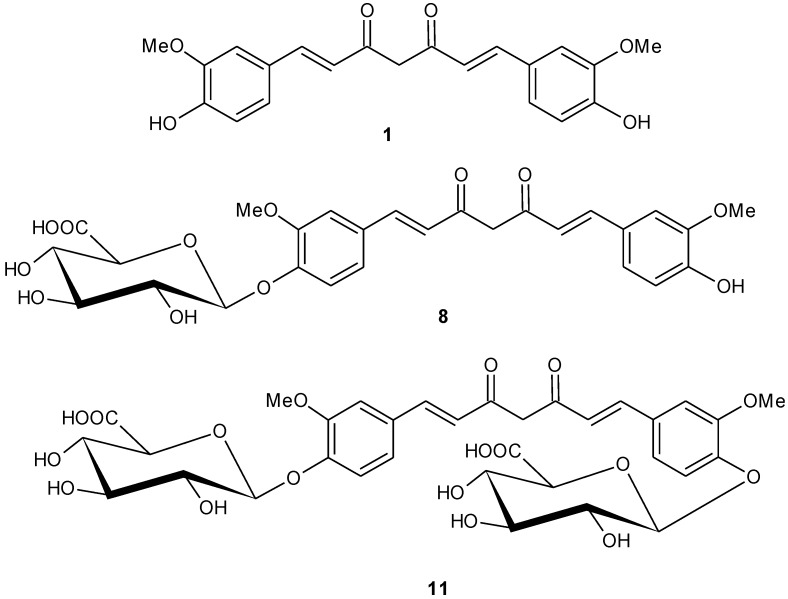
Chemical structures of Curcumin (**1**), curcumin monoglucuronide (**8**) and curcumin diglucuronide (**11**).

The access of alternative synthetic approaches in the literature on the synthesis of the same molecule could open up a number of choices in the scientific community in case they wish to elaborate the study in the same scientific field. The chemical synthesis of curcumin monoglucuronide was reported recently by Bornmann *et al*. [21], however we have developed an alternative synthetic approach to synthesize curcumin monoglucuronide in gram quantity. Our practical synthesis of curcumin monoglucuronide (Figure 1) in gram quantity has not yet been reported.

Meanwhile, analytical data for curcumin diglucuronide (Figure 1) and its acetylated intermediate were reported [21] but so far there are no experimental details accessible from the literature for synthesizing these two molecules. We are the first to report herein the experimental details on synthesizing curcumin diglucuronide and its intermediate acetylated curcumin diglucuronide.

Oxidative stress is the common phenomenon that causes damage in our body system due to the generation of free radicals. Negligence of these stresses could exacerbate free radical damage. It is reported [22,23] that free radicals could damage DNA forming as a main product 8-oxo-guanine [23] and it may lead to the incidence of cancer [22,23]. Anti-oxidants [24,25] generally scavenge free radicals [24], stabilize them from free radical damage and help in normalizing health disorders [25]. Anti-oxidants are also recommended as chemopreventive agents for cancer [26].

Curcumin is well recognized as an anti-oxidant and reported to have free radicals scavenging activity [27] that recommends its application as an anti-cancer agent [1]. However, it is not clear whether curcumin retains its anti-oxidant activity after it is orally administered or after bioconversion *in vivo* into its metabolites, curcumin glucuronides, that may still retain the anti-oxidant activity inside the body system. To find a plausible explanation, we focused on synthesizing and studying the anti-oxidant activity of curcumin glucuronides in focused manner with respect to curcumin and the results are elaborated in this report. This study clearly demonstrates that curcumin monoglucuronide shows 10 fold less anti-oxidant activity and curcumin diglucuronide is not at all noticeably effective as an anti-oxidant in comparison to anti-oxidant activity of curcumin (by DPPH). It is concluded that glucuronide metabolites of curcumin, namely, curcumin monoglucuronide and curcumin di-glucuronide are not active components exhibiting anti-oxidant activity of curcumin after its oral administration.

Thus our studies show that the metabolic conversion of curcumin into the two glucuronides (**8**) and (**11**) render it into essentially inactive anti-oxidant compounds that may not exert the necessary anti-oxidant action in disease states as delineated earlier. This observation combined with the findings of others that the curcumin glucuronides (**8**) and (**11**) are ineffective as NFκB inhibitors [21] as well as very poorly cytotoxic to cancer cell lines [20,21] support the conclusion that unmodified curcuminoids are the pharmacologically active agents while their glucuronidated biotransformed products are not.

## 2. Experimental

### 2.1. Reagents, Instrumentation

All chemicals were purchased either from Spectrochem, India, Acros Organics, India or Aldrich, USA and used directly without further purification. Solvents (hexane, tetrahydrofuran, ethylacetate, acetone, dichloromethane, chloroform, methanol, and ethanol) were purchased from commercial suppliers in India and were dried over sodium metal (hexane and tetrahydrofuran), over potassium carbonate (ethylacetate and acetone), over calcium chloride (dichloromethane and chloroform) and over magnesium turning (methanol and ethanol) before use. HPLC grade *N*,*N*-dimethyl formamide (DMF) was purchased from Spectrochem, India and used as received. Reaction was monitored by thin layer chromatography (TLC) on TLC silica gel F_254_ (Merck Specialities Private Ltd., Mumbai, India) and visualized either under UV (254 nm) or by charring with 5% sulfuric acid in methanol. Melting points were checked by using LABINDIA MR-VIS (visual melting range) apparatus and were uncorrected. ^1^H and ^13^C-NMR spectra were recorded on a Varian 300 MHz NMR Instrument. Chemical shifts were reported in ppm relative to TMS as reference standard. Coupling constants were presented in Hertz and splitting pattern were assigned as s, singlet; d, doublet; t, triplet; m, multiplet, and bs, broad singlet. The purity of compounds was verified by analytical HPLC. HPLC analyses were performed in Shimadzu Labsolutions HPLC System. Analytical reversed phase C_18_ column (ThermohypersilBDS, UK) (250 mm × 4.6 mm, 5 µm) was used with a gradient where mobile phase A was water:acetonitrile:acetic acid (8.5:1.5:0.005) and mobile phase B was methanol. Flow rate was 1.0 mL/min with UV detection at 420 nm. Mass spectra were recorded on Thermo-Fisher LCQ Advantage Max (LAM-10234) ion trap mass spectrometer. Column chromatography was performed on silica gel (60–120 mesh) purchased from commercial supplier, India.

#### 2.1.1. Synthesis of Methyl [1-*O*-(4′-formyl-2′-methoxyphenyl)-2,3,4-tri-*O*-acetyl-β-d-glucopyranosiduronate) (**4**)

Methyl 1-bromo-2,3,4-tri-*O*-acetyl-α-d-glucopyranosiduronate (**2**) (27g, 68 mmol) and vanillin (**3**) (11 g, 72.4 mmol) were dissolved in 120 mL of chloroform and then 120 mL of 1N sodium hydroxide solution and tetrabutyl ammonium bromide (27g, 72.4 mmol) were added. The reaction mixture was stirred at 50 °C for 4 h. It was cooled to RT and diluted with 250 mL of chloroform. Organic layer was washed with water (100 mL × 2), cold 1N hydrochloric acid solution (100 mL × 3), water (100 mL × 2) and brine solution (100 mL × 1), respectively. It was dried over sodium sulfate, filtered and concentrated to obtain 17 g of crude material. The crude material was purified by column chromatography over silica gel (400 g). Elution with 1:1 hexane-ethyl acetate afforded compound **4** as white powder. Yield 9.6 g (28%), R_f_ 0.40 (1:1 hexane-ethyl acetate). ^1^H-NMR (CDCl_3_): δ 9.81 (s, 1H, C*H*O), 7.38–7.35 (m, 2H), 7.19 (d, 1H, *J =* 7.2 Hz), 5.31–5.23 (m, 3H), 5.15 (d, 1H, *J =* 6.6 Hz, H-1′), 4.14 (d, 1H, *J =* 8.1 Hz, H-4’), 3.82 (s, 3H, -C6H3OC*H*_3_), 3.66 (s, 3H, -COOC*H*_3_), 2.01. 1.97. 1.93 (3s, 9H, OCOC*H*_3_). ^13^C-NMR (CDCl_3_): δ 191.3 (-*C*HO), 170.3 (*C*OOCH_3_), 169.6, 169.4, 167.1, 151.1, 151.0, 133.1. 125.7, 118.7, 110.8, 99.7, 77.8, 72.8, 71.7, 71.0, 69.2, 56.3, 53.2, 20.82, 20.8, 20.7 (MS-APCI): 485.83 (M + NH_4_)^+^(C_21_H_24_O_12_.NH_4_^+^ requires 486.4465).

#### 2.1.2. 4-(4-hydroxy-3-methoxyphenyl)-3-buten-2-one (**5**)

Vanillin (**3**) (20 g, 131.6 mmol) was dissolved in 120 mL of acetone and then a solution of sodium hydroxide (6g, 148 mmol) in water (80 mL) was added. The reaction mixture was stirred at 0–10 °C for 1 h. Ice-bath was removed and it was stirred at RT for 48 h. The reaction mixture was evaporated to dryness and then acidified with aqueous 1 N hydrochloric acid solution. Precipitated solid was filtered, washed with water (100 mL × 3) and dissolved in 200 mL of dichloromethane. The solution was dried over sodium sulfate, filtered and evaporated. The crude product was stirred with 100 mL of ethyl acetate for 30 min. Precipitated solid was filtered and washed with ethyl acetate (50 mL × 2) and dried under high vacuum to obtain titled compound (**5**) as yellow powder. Yield 18 g (71%). R_f_ 0.80 (1:1 hexane-ethyl acetate). ^1^H-NMR (CDCl_3_): δ 7.42 (d, 1H, *J =* 16.2 Hz, H-4), 7.06–7.01 (m, 2H), 6.90 (d, 1H, *J =* 8.1 Hz), 6.55 (d, 1H, *J =* 16.2 Hz, H-3), 3.88 (s, 3H, -C_6_H_3_OC*H*_3_), 2.33 (s, 3H, H-1). ^13^C-NMR (CDCl_3_): δ 198.9, 148.5, 147.1, 144.2, 126.9, 125.0, 123.7, 115.1, 109.5, 56.1, 27.4. MS (APCI): 193.05 (M + H)^+^, (C_11_H_13_O_3_ requires 193.2189).

#### 2.1.3. 5-Hydroxy-1-(4-hydroxy-3-methoxyphenyl)-1,4-hexadien-3-one (**6**)

To a mixture of tetrahydrofuran (50 mL) and ethanol (5 mL, 87 mmol), sodium metal (2 g, 87 mmol) was added and it was refluxed at 95–100 °C until all sodium metal was consumed (~4 h). A solution of compound **5** (5 g, 26 mmol) in tetrahydrofuran (25 mL) and ethyl acetate (25 mL) was then added dropwise (~10 min). After completion of addition, the reaction mixture was stirred at 95–100 °C for 3 h. It was cooled to RT and diluted with 100 mL of ethylacetate. Organic layer was washed with cold 1N hydrochloric acid solution (100 mL × 3), water (50 mL × 1) and brine solution (50 mL × 1), respectively. The crude material was purified by column chromatography over silica gel (60 g). The column was eluted with 2:1 hexane-ethyl acetate and the desired fractions were collected, concentrated and dried under high vacuum to afford compound **6** as yellow powder. Yield 2.8 g (46%). R_f_ 0.75 (1:1 hexane-ethyl acetate). ^1^H-NMR (CDCl_3_):1H-NMR (DMSO-d_6_): δ 9.66 (s, 1H), 7.51 (d, 1H, *J =* 15.9 Hz, H-6), 7.31 (d, 1H, *J =* 1.8 Hz), 7.14–7.11 (m, 1H), 6.83 (d, 1H, *J =* 8.1 Hz), 6.65 (d, 1H, *J =* 16.2 Hz, H-5), 5.84 (s, 1H), 3.84 (s, 3H, -C_6_H_3_OC*H*_3_), 2.13 (s, 3H, H-1). ^13^C-NMR (DMSO-d_6_): δ 196.8, 178.3, 149.2, 148.0, 140.3, 126.4, 123.0, 119.7, 115.7, 111.2, 100.6, 55.7, 26.5. MS (APCI): 232.91 (M−H)^−^ (C_13_H_13_O_4_ requires 233.2400).

#### 2.1.4. Mono-[methyl 2,3,4-tri-*O*-acetyl-β-d-glucopyranosiduronate]-curcumin (**7**)

Compound **5** (1.5g, 6.4 mmol) was dissolved in DMF (50 mL) and then added 0.7 g (9.6 mmol) of boric anhydride. It was stirred at 75–80 °C for 30 min and then added 7 mL (25.6 mmol) of tri-*s*-butyl borate and stirred for additional 1h. A solution of compound **4** (3 g, 6.4 mmol) in DMF (10 mL) was then added followed by 0.3 mL (2.6 mmol) of *n*-butyl amine. The reaction mixture was stirred at 75–80 °C for 4 h. It was cooled to 50–55 °C, 10% aqueous acetic acid (100 mL) was then added and stirred for 1 h. After cooling to RT, 100 mL of water was added and stirred for 30 min and precipitated solid was collected after decanting the aqueous solution. Solid material was dissolved in 150 mL of ethyl acetate and washed with cold 1N hydrochloric acid solution (30 mL × 3), water-brine (4:1) (50 mL × 3) and brine solution (50 mL × 1) respectively. Organic layer was dried over sodium sulfate, filtered and evaporated. The crude material was purified by three consecutive crystallizations. Crude material was dissolved in 150 mL of ethyl acetate and then 100 mL of hexane was added portion-wise and stirred at RT for 30 min. Precipitated material was collected by filtration. It was precipitated again by dissolving in 20 mL of chloroform and adding 60 mL of hexane. It was repeated one more time and the precipitated yellow solid was collected by filtration and dried under high vacuum to afford titled compound **7** as yellow powder. Yield: 3.2 g (yellow solid) (73%). R_f_ = 0.35 (1:1, hexane:ethyl acetate). ^1^H-NMR (CDCl_3_): δ 7.57 (d, 1H, *J =* 15.6 Hz), 7.54 (d, 1H, *J =* 15.6 Hz), 7.14–7.02 (m, 5H), 6.92–6.89 (m, 1H), 6.50 (d, 1H, *J =* 15.9 Hz), 6.46 (d, 1H, *J =* 15.9 Hz), 5.8 (s, 1H), 5.38–5.28 (m, 3H), 5.09 (d, 1H, *J =* 6.0 Hz, H-1), 4.15 (d, 1H, *J =* 7.8 Hz, H-4), 3.91 (s, 3H), 3.84 (s, 3H), 3.74 (s, 3H), 2.09, 2.06 and 2.06 (3s, 9H). ^13^C-NMR (CDCl_3_): δ 184.4, 182.4, 170.4, 169.7, 169.6, 167.2, 150.9, 148.3, 147.5, 147.1, 141.3, 139.7, 132.0, 127.7, 123.7, 123.2, 121.8, 121.7, 120.1, 115.2, 111.8, 110.0, 101.8, 100.4, 77.8, 72.7, 71.9, 71.2, 69.4, 56.2, 56.1, 53.2, 20.9, 20.8 and 20.7. MS (APCI): 684.84 (M+H)^+^ (C_37_H_49_O_5_ requires 684.6406).

#### 2.1.5. Mono-(β-d-glucopyranosiduronic acid)-curcumin (curcumin monoglucuronide) (**8**)

Compound **7** (2.75 g, 4 mmol) was dissolved in methanol (50 mL) and then 22 mL of 0.3 N sodium methoxide solution was added. The reaction mixture was stirred at RT for 4 h and then 22 mL of water was added. It was stirred at RT for another 14 h and then acidified with Dowex (H^+^) resin. The solution was filtered, concentrated and dried under high vacuum. The crude product was purified by column chromatography on silica gel (80 g). Column was eluted with 4:1, 2:1 and 1:1 chloroform–methanol. Desired fractions were collected and concentrated and dried under high vacuum for 2 h to obtain the desired compound **8** as yellow powder. Yield: 1.25 g (57%). MP 158–160 °C, R_f_ = 0.65 (2:1 CHCl_3_-MeOH). HPLC purity 96%, retention time: 29.70 min. ^1^H-NMR (DMSO-d_6_): δ 9.8, (bs, COOH), 7.54 (d, 2H, *J =* 15.9 Hz), δ 7.32 (d, 2H, *J =* 17.4 Hz), 7.22 (d, 1H, *J =* 17.4 Hz), 7.15–7.09 (m, 2H), 6.85–6.80 (m, 2H), 6.07 (s, 1H), 5.18 (bs, 2H), 3.99–3.91 (m, 2H), 3.81 (s, 6H). ^13^C-NMR (DMSO-d_6_): δ 184.7, 183.1, 170.8, 150.0, 149.8, 148.6, 148.5, 141.8, 140.5, 129.7, 127.0, 123.9, 123.3, 121.8, 116.4, 115.6, 111.9, 101.9, 99.8, 76.6, 76.1, 73.5, 72.0, 56.34 and 56.3. MS (APCI): 543.12 (M−H)^−^ (C_27_H_27_O_12_ requires 543.4962), Anal Calcd for C_27_H_28_O_12_: C_found_: 59.56, H_found_: 4.82. (C_calcd_: 59.56, H_calcd_: 5.18).

#### 2.1.6. Bis-[methyl 2,3,4-tri-*O*-acetyl-β-d-glucopyranosiduronate]-curcumin (**10**)

Acetyl acetone (**9**) (0.4mL, 4.1 mmol) was dissolved in DMF (50 mL) and 0.3 g (4.1 mmol) of boric anhydride was added. The reaction mixture was stirred at 75–80 °C for 1 h, 4.6 mL (17.1 mmol) of tri-*s*-butyl borate was then added and stirred for additional 1 h. A solution of compound **4** (4 g, 8.55 mmol) in DMF (10 mL) was then added followed by 0.2 mL (1.7 mmol) of n-butyl amine. The reaction mixture was stirred at 75–80 °C for 3 h. It was cooled to 50–55 °C and 10% aqueous acetic acid (100 mL) was then added and stirred at 50–55 °C for 1 h. After cooling to RT, water (100 mL) was added and stirred for 30 min. A solid was precipitated. The aqueous layer was decanted and the solid was washed twice with water (20 mL × 2). Precipitated solid was dissolved in ethyl acetate (150 mL). Organic layer was washed with cold 1 N hydrochloric acid solution (30 mL × 3), water-brine (4:1) (50 mL × 3) and brine solution (50 mL × 1), respectively. Organic layer was dried over sodium sulfate and evaporated. The crude material was purified by column chromatography on silica gel (200 g). Column was eluted with 1:1 and 2:1 ethyl acetate-hexane. Desired fractions were pooled, concentrated and dried under high vacuum to afford compound **10** as yellow powder. Yield: 2.4 g (59%). R_f_ = 0.2 (1:1 hexane-ethyl acetate). ^1^H NMR (CDCl_3_): δ 7.51 (d, 2H, *J =* 15.6 Hz), 7.10–7.01 (m, 6H), 6.46 (d, 2H, *J =* 15.9 Hz), 5.8 (s, 1H), 5.32–5.22 (m, 6H), 5.05 (d, 2H, *J =* 6.6 Hz, H-1′), 4.11 (d, 2H, *J =* 6.9 Hz, H-4’), 3.80 (s, 6H), 3.70, 3.69 (2s, 6H), 2.04, 2.03, 2.02, 2.01, 2.00 and 1.99 (6s, 18H). ^13^C-NMR (CDCl_3_): δ 183.3, 170.3, 169.6, 169.5, 167.1, 150.9, 147.6, 140.2, 131.9, 123.7, 121.9, 120.1, 111.9, 101.9, 100.4, 76.9, 72.8, 71.9, 71.2, 69.4, 56.3, 53.1, 20.8 and 20.7. MS (APCI): 1000.74 (M)^+^ (C_47_H_52_O_24_ requires 1000.9013).

#### 2.1.7. Bis-(β-d-glucopyranosiduronic acid)-curcumin (curcumin diglucuronide) (**11**)

Compound **10** (0.5 g, 0.5 mmol) was taken in 20 mL of methanol and 5 mL of 0.3N sodium methoxide solution was then added. The reaction mixture was stirred at RT for 8 h and then 5 mL of water was added and stirred at RT for another 18 h. It was then acidified with Dowex (H^+^) resin, filtered, concentrated and dried under high vacuum. The crude product was subjected to crystallization. Crude mixture was taken in 5 mL of methanol and 25 mL of ethyl acetate was added and stirred for 30 min. Precipitated yellow solid was collected by filtration and washed with ethyl acetate (10 mL × 2). It was dried under high vacuum to obtain compound (**11**) as yellow powder. Yield: 0.15g, R_f_ = 0.15 (2:1 CHCl_3_-MeOH). HPLC purity 82%, retention time: 19.59 min. MS (APCI): 719.06 (M−H)^−^ (C_33_H_35_O_18_ requires 719.6203).

### 2.2. Antioxidant Assay by DPPH [28,29]

The assay mixture contains 1.5 mL of 0.1mM DPPH methanolic solution, dimethyl sulfoxide (DMSO) solutions of various concentrations of the material and methanol in a total volume of 3 mL. Blanks (1.5 mL of methanol and 1.5 mL of DPPH solution) and controls (0.5 mL of curcumin solution in DMSO and 2.5 mL of methanol) were also taken. The mixture was incubated at 37 °C for 30 min. The reduction of absorbance was measured spectrophotometrically at 516 nm and the result is summarized in Table 1.

The free radical scavenging activity is expressed as SC_50_ values, the concentration of the sample required for 50% of the free radical to be scavenged. It is calculated according to the following equation

(1) $$\text{\% Scavenging}=\frac{\text{Ac }-\text{ At}}{\text{Ac}}\text{ x }100\;$$

where, Ac = absorbance of control, At = absorbance of test solution.

**Table 1:** Results of DPPH assay (values represent means ± SD).

Compounds	Concentration (µg/mL)	DPPH Scavenging Activity (%)	SC _50_ (μg/mL)
Curcumin (**1**)	3.33	72.07 ± 0.19	1.58
	1.67	50.14 ± 0	
	0.83	27.79 ± 1.93	
	0.42	21.8 ± 0.38	
	0.21	10.08 ± 0	
Curcumin monoglucuronide (**8**)	33.33	79.5 ± 0.44	15.61
	16.67	54.5 ± 0.21	
	8.33	23.6 ± 0.44	
	4.17	6.3 ± 0.22	
Curcumin diglucuronide(**11**)	333.33	29.34 ± 0.62	SC_50_ was not obtained. Inhibition of 29.77% was obtained at 333 μg/mL
	166.67	19.5 ± 0.21	
	83.33	9.68 ± 0.62	
	41.67	6.94 ± 0	
	20.83	2.89 ± 0.82	

SC_50_ is the concentration at which 50% scavenging of DPPH is obtained. The lower the SC_50_, the better the efficacy, SD is standard deviation.

#### Antioxidant Assay by Oxygen Radical Absorbance Capacity (ORAC) [30]

Twenty-five microliters of different concentrations of the sample was pipetted into each well followed by 150 μL of 10 × 10^−2^ M (final conc.) AAPH reagent (2,2’-Azobis-(2-amidinopropane) dihydrochloride) made in 75 mM potassium phosphate buffer (pH 7.4). Then, 150 μL of disodium fluorescein dye (final conc. 4.8 × 10^−7^ M) was added and mixed before the initial reading (f_0_) was taken. Fluorescence reading was taken on Fluostar Optima Microplate Reader at 485/520 nm after every 1 min for 35 min (f_1_……..f_35_). Twenty-five microliters of phosphate buffer (75 mM) was pipetted in the blank instead of antioxidant. Trolox standard from 12.5 to 200 μM was also prepared and used. The number of wells being used in the experiment should not exceed 20 to reduce the error due to time lag. Difference between duplicates also occurs due to the time lag and to avoid this, the experiment was repeated thrice with % CV not more than 15.

The final ORAC values were calculated by using a quadratic regression equation (*Y* = a + b *X* + c *X*^2^) between the trolox concentration (*Y*) (μM) and the net area under the Fluorescence decay curve (*X*) and were expressed as micromoles of trolox equivalents per liter or per gram of sample (μmol TE/g or μmol TE/L).

(2) The Area under curve (AUC) = (1 + f_1_/f_0_ + f_2_/f_0_ + …. + f_35_/f_0._)

where f_0_ is the initial fluorescence reading at 0 min and f_1_ is the fluorescence reading after 1 min.

The data were analyzed by applying Equation (2). The net AUC was obtained by subtracting the AUC of the blank from that of the sample. The value calculated using the net AUC of the sample and the quadratic regression equation was divided by the concentration of the sample in g/L. The final value obtained is the ORAC value of the sample expressed as μmol TE/g and the result is shown in Table 2.

**Table 2:** Results of ORAC assay.

Compounds	Sample (mg/L)	ORAC Value (µmol TE/g)	Average ORAC Value (µmol TE/g)	SD
Curcumin (**1**)	0.781	15,830.010	14,981.34	298.41
	1.563	15,164.094		
	3.125	14,981.707		
	6.250	14,479.844		
	12.500	14,451.057		
Curcumin monoglucuronide (**8**)	3.125	6891.506	6891.349	340.893
	6.250	7132.319		
	12.500	6650.224		
Curcumin diglucuronide(**11**)	6.250	2449.353	2502.489	175.149
	12.500	2425.833		
	25.00	2373.853		
	50.00	2760.917		

The higher the ORAC value, the better the efficacy. SD is standard deviation. The ORAC average and SD values were calculated using a regression equation between the Trolox (standard) concentration and the net area under the curve (AUC). The sample net AUC should correspond to the Trolox net AUC.Thus, the concentrations of the test sample that falls under the range of net AUC for Trolox are chosen for the calculation of the average ORAC value.

## 3. Results and Discussion

### 3.1. Synthesis

**Scheme 1:**
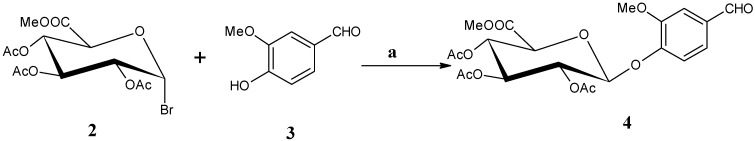
Synthesis of methyl [1-*O*-(4′-formyl-2′-methoxyphenyl)-2,3,4-tri-*O*-acetyl-β-d-glucopyranosiduronate] (**4**). Reagents: (a) CHCl_3_, NaOH, (Bu_4_N)^+^Br^−^, 50 °C, 4 h.

**Scheme 2:**
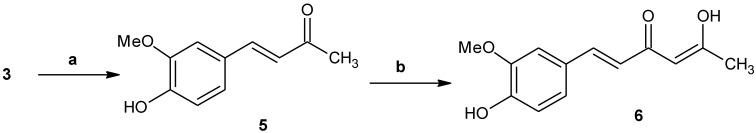
Synthesis of 5-Hydroxy-1-(4-hydroxy-3-methoxyphenyl)-1,4-hexadien-3-one (**6**). Reagents: (a) Acetone, NaOH, RT, 18h, (b) Na, EtOH, EtOAc, reflux, 6 h.

**Scheme 3:**
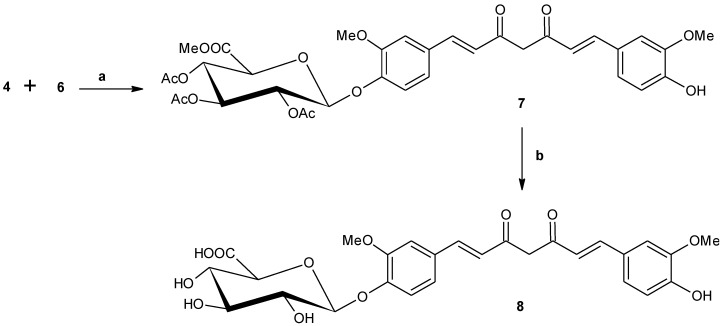
Synthesis of mono-(β-d-glucopyranosiduronic acid)-curcumin (curcumin-monoglucuronide) (**8**). Reagents: (a) B_2_O_3_, (sBuO)_3_B, *n*-BuNH_2_, DMF, 75–80 °C, 6 h, (b) (i) NaOMe, MeOH, H_2_O, RT, 24 h;(ii) Dowex (H^+^), RT, 30 min.

The synthesis of curcumin monoglucuronide was performed in three stages, as described in Scheme 1, Scheme 2 and Scheme 3.

First stage was involved in the synthesis of key intermediate methyl [1-*O*-(4′-formyl-2′-methoxyphenyl)-2,3,4-tri-*O*-acetyl-β-d-glucopyranosiduronate] (**4**) (Scheme 1) by condensing known methyl 1-bromo-2,3,4-tri-*O*-acetyl-α-d-glucopyranosiduronate (**2**) [31,32] with vanillin (**3**) in presence of phase transfer catalyst and provided 28% yield. There are few reports [21,33] on the synthesis of same molecule by using either expensive reagents [33] or by using metal catalyst [21]. Our approach consists of eco-friendly, inexpensive simple phase transfer condition, which provided a reasonable yield.

In the second stage, target synthon 5-hydroxy-1-(4-hydroxy-3-methoxyphenyl)-1,4-hexadien-3-one (**6**) was synthesized in two consecutive chemical reactions. So far the synthesis of compound **6** was reported in one step by reacting vanillin (**3**) with acetyl acetone [21,34,35]. This is the first report consisting of an alternative synthetic strategy involving two steps to synthesize compound **6** and it is outlined in Scheme 2. This strategy was developed in order to have alternative access for obtaining good quantity of the final target molecule. In this approach, vanillin (**3**) was first condensed with acetone in the presence of aqueous sodium hydroxide to provide compound **5**. Recently, the synthesis of compound **5** was reported [36] and purified by column chromatography. We have isolated compound **5** by crystallization with 71% yield. Compound **5** was then refluxed with sodium and ethanol in tetrahydrofuran followed by addition of ethyl-acetate to afford the crude compound **6**. Purification of crude product by column chromatography on silica gel provided compound **6** with 46% yield. The structures of both compounds **5** and **6** were confirmed by their ^1^H, ^13^C-NMR spectra and from their mass spectra.

In the third stage, the synthesis of desired molecule, curcumin monoglucuronide (**8**) was achieved by two consecutive reactions, as described in Scheme 3. The first reaction is the usual coupling reaction for getting curcumin moiety following the pioneer work of Pabon *et al*. [37] with minor modification. Generally, ethyl acetate is used as solvent for the formation of curcumin skeleton, however, it was found that the more polar solvent, *N*,*N*-dimethyl formamide (DMF) is also suitable for the same reaction. Compound **6** was treated with boric anhydride in DMF at 75 to 80 °C for 1 h; tri*s*-secbutyl borate was then added and stirred for another 1 h. Compound **4** was dissolved in DMF and added into the reaction mixture followed by addition of n-butyl amine. The reaction mixture was then stirred at 75 to 80 °C for 4 h. The temperature was maintained at 50–55 °C, and then a 10% aqueous acetic acid solution was added and finally it was stirred for an additional 1 h. Product **7** was isolated from crude mixture by crystallization with 73% yield. The structure of compound **7** was assigned from its ^1^H, ^13^C-NMR spectra and from mass spectrum. Deacetylation of compound **7** using sodium methoxide in methanol followed by *in situ* hydrolysis of methyl ester by adding water followed by acidification with Dowex (H^+^) resin afforded the crude compound **8**. Purification by column chromatography over silica gel provided the desired curcumin monoglucuronide (**8**) with 57% yield. Compound **8** was characterized by its ^1^H, ^13^C-NMR spectra, and mass spectrum as well as from its elemental analysis. The purity of this molecule was verified by analytical HPLC on reversed phase C_18_ column and obtained with 96% HPLC purity (supporting information p-20).

**Scheme 4:**
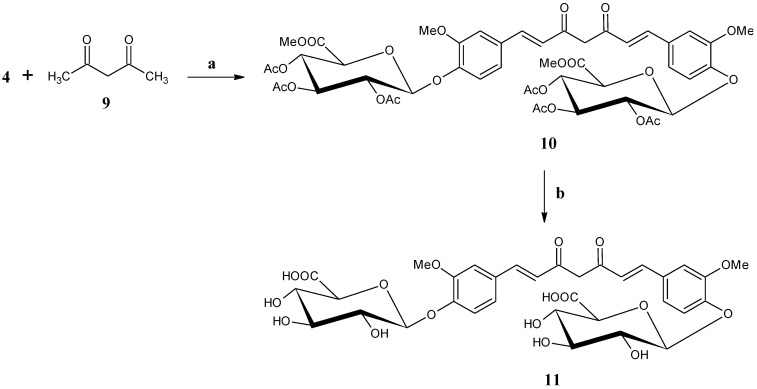
Synthesis of Bis-(β-d-glucopyranosiduronic acid)-curcumin (curcumin diglucuronide) (**11**). Reagents: (a) B_2_O_3_, (sBuO)_3_B, n-BuNH_2_, DMF, 75–80 °C, 6 h, (b) (i) NaOMe, MeOH-CH_2_Cl_2_, H_2_O, RT, 18 h, (ii) Dowex (H^+^), RT, 30 min.

So far, except the analytical data for acetylated curcumin diglucuronide and curcumin diglucuronide [21], no experimental details are available in the literature. We are the first to report herein the experimental details of the synthesis of acetylated curcumin diglucuronide (**10**) and curcumin diglucuronide (**11**) (Scheme 4).

The usual coupling reaction was used for getting curcumin moiety following the pioneer work of Pabon *et al*. [37] with minor modification as described in the synthesis of compound **7**. The synthesis of acetylated curcumin diglucuronide (**10**) was achieved by reacting acetyl acetone (**9**) with boric anhydride in DMF in the presence of tris sec-butyl borate followed by addition of compound **4** in DMF and n-butyl amine. The reaction mixture was then stirred at 75 to 80 °C for 4h. The temperature was then maintained at 50–55 °C and added 10% aqueous acetic acid solution and stirred for an additional 1 h. The acetylated curcumin diglucuronide (**10**) was purified from crude reaction mixture by column chromatography and obtained with 59% yield. The structure of compound **10** was assigned from its ^1^H, ^13^C-NMR spectra, and mass spectrum. Deacetylation of compound **10** in the presence of sodium methoxide in methanol followed by addition of water for *in situ* hydrolysis of methyl ester followed by acidification with Dowex (H^+^) resin provided crude curcumin diglucuronide (**11**). Purification by crystallization was attempted, however we could only achieve 82% HPLC purity (Supporting Information p-25) of the desired curcumin diglucuronide (**11**). It was not further purified by preparative HPLC, as analytical data already exist in the literature [21]. Compound **11** having HPLC purity 82% was used for anti-oxidant activity study in order to evaluate its biological function with its existing purity state.

### 3.2. Anti-Oxidant Studies

The strength of anti-oxidants is determined by measuring its free radical scavenging ability. Numerous methods are generally used to measure the capacity of anti-oxidants; however we have focused on two methods described as follows.

#### 3.2.1. Antioxidant Assay by DPPH Scavenging Method [28,29]

The free radical scavenging activity is determined based on the interaction of stable free radical 1,1-diphenyl-2-picryl hydrazyl radical (DPPH) [28,29] with antioxidant in organic/aqueous organic media resulting in bleaching of the DPPH as it gets quenched by the analytes. The concentration of antioxidant in the test solution was determined by the decrease of absorbance of DPPH compared to blank and was measured spectrophotometrically at 516 nm.

#### 3.2.2. Antioxidant Assay by Oxygen Radical Absorbance Capacity (ORAC) [30]

The Oxygen Radical Absorbance Capacity (ORAC) assay [30] depends on the free radical damage to a fluorescent probe through the change in its fluorescence intensity. The change of fluorescence intensity is an index of the degree of free radical damage. In presence of antioxidants, the free radical damage is inhibited and this is reflected in the protection against the change of probe fluorescence, which is the measure of its antioxidant capacity against the free radical.

**Figure 2:**
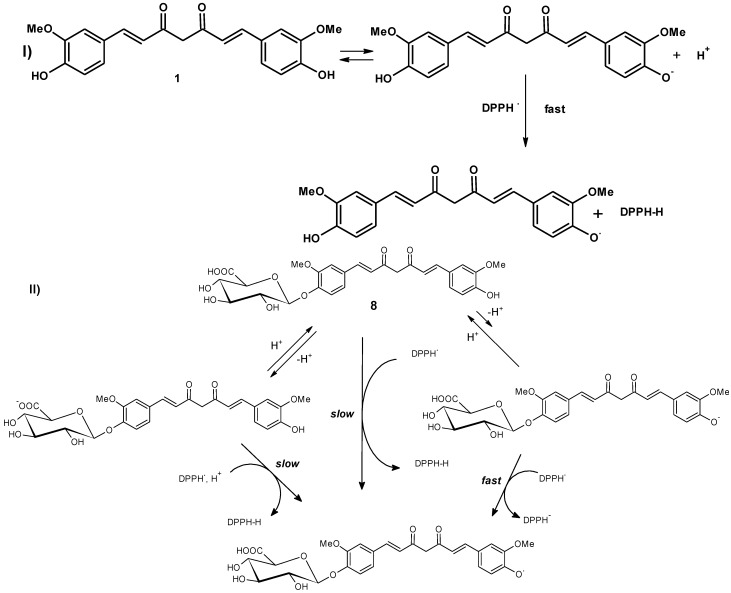
Possible sequential proton loss electron transfer (SPLET) pathway of (**I**) curcumin (**1**) and (**II**) curcumin monoglucuronide (**8**).

The interesting observation that the anti-oxidant activity of curcumin monoglucuronide (**8**) falls precipitously by several folds in DPPH method is indicative of the fact that the anti-oxidant action of curcumin is mediated more by the SPLET (sequential proton loss electron transfer) mechanism than by HAT (hydrogen atom transfer) pathway [38,39]. In SPLET pathway, curcumin is prone to form anion in an equilibrium, which then transfers an electron to DPPH. Tendency to form such phenolic anion in the monoglucuronide of curcumin (**8**) is very low due to the presence of glucuronic acid moiety with an acidic carboxyl group in its structure and hence SPLET pathway becomes insignificant [40] as represented in Figure 2.

The presence of -COOH in the monoglucuronide, with an acidity ~10^5^ times higher than the phenolic –OH will definitely reduce the phenoxide concentration that would be needed for effective radical scavenging functioning of the curcumin monoglucuronide.

Curcumin diglucuronide (**11**) (not shown in figure) is thus not effective as radical scavenger.

In the ORAC method, which estimates the radical chain-breaking capacity of the anti-oxidant primarily acting through the HAT pathway, the decrease in the anti-oxidant capacity of the glucuronides of curcumin (**8**) and (**11**) is more gradual [41] and consistent with progressive blockage of the phenolic groups with glucuronic acid moiety. The decrease in ORAC values is consistent with the fact that the anti-oxidant activity of curcumin in ORAC systems are dependent on the phenolic –OH than the enolic –OH [40] since the bond dissociation energy (BDE) is less for the former by ~5 kcal/mol according to DFT calculations [42].

## 4. Conclusions

Gram quantity of curcumin monoglucuronide (**8**) and milligram quantity of curcumin diglucuronide (**11**) were synthesized using an alternative synthetic approach. This report illustrates for the first time the experimental details of synthesis of acetylated curcumin diglucuronide (**10**) and curcumin diglucuronide (**11**) that have so far not yet appeared in the literature [21]. The HPLC purity of compounds **8** and **11** were verified by analytical HPLC and found to have purities of 96% and 82%, respectively. Curcumin monoglucuronide (**8**) was characterized not only with its ^1^H and ^13^C-NMR and mass spectra but also by its elemental analysis. This synthetic approach illuminates alternative access for practical synthesis of curcumin monoglucuronide in gram quantity. Synthesized curcumin monoglucuronide (**8**) and curcumin diglucuronide (**11**) were studied to determine their anti-oxidant activity in comparison with curcumin. Their anti-oxidant activity was measured by DPPH scavenging method and by Oxygen Radical Absorbance Capacity (ORAC) assay. The biological results demonstrated that curcumin monoglucuronide (**8**) eventually exhibited 10 fold less anti-oxidant activity and curcumin diglucuronide (**11**) was not at all comparable in respect to the anti-oxidant activity of curcumin.

In curcumin monoglucuronide (**8**) and curcumin diglucuronide (**11**), the enolic hydroxyl group of the predominant enol tautomer is free. This probably contributes very little to the anti-oxidant capacity of curcumin as assessed by DPPH method. This is in accordance with the conclusions arrived at by Liu *et al*. [43]. The absence of anti-oxidant activity, anti-cancer-activity and absence of NF-κB inhibiting activity of these curcumin glucuronides **8** and **11** also lead to the conclusion that these glucuronide metabolites of curcumin may not play any significant role in the pharmacological activity of curcumin. Despite the poor anti-oxidant activity of glucuronide metabolites of curcumin, curcumin still displays its pharmacological activity after oral administration. Thus, the pharmacological effects of curcumin may not be entirely due to its antioxidant activity, but could also be due to its pleiotropic pharmacology, which might be operational when interacting with its molecular targets. Alternatively, other reductive metabolites such as tetrahydrocurcumin might play a significant role in mediating the effects of curcumin.

## Supporting Information

^1^H, ^13^C-NMR spectra and mass spectra of compounds **4**, **5**, **6**, **7**, **8** and **10**. Elemental analysis data of compound **8**. Mass spectrum of compound **11**. HPLC chromatograms of compounds **8** and **11**.

## List of Abbreviations

DPPH	1,1-Diphenyl-2-picryl hydrazyl
ORAC	Oxygen Radical Absorbance Capacity
LC-MS	Liquid chromatography-mass spectrometry
HPLC	High-performance liquid chromatography
(Bu_4_N)^+^Br^−^	Tetrabutyl ammonium bromide
RT	Room Temperature
B_2_O_3_	Boric anhydride
(sBuO)_3_B	tri-*s*-butyl borate
*n*-BuNH_2_	*n*-Butyl amine
NaOMe	Sodium methoxide
MS (APCI)	Mass Spectrometry (Atmospheric pressure chemical ionization)
